# Effect of intra-abdominal volume increment on kidneys in minipigs with intra-abdominal hypertension after hemorrhagic shock and resuscitation

**DOI:** 10.1186/2054-9369-1-4

**Published:** 2014-04-15

**Authors:** Zheng-gang Wang, Hao Tan, Lian-yang Zhang, Dao-cheng Liu, Hua-liang Xiao, Wen-hua Du

**Affiliations:** State Key Laboratory of Trauma, Burns and Combined Injury, Trauma Center, Institute of Surgery Research, Daping Hospital, Third Military Medical University, Chongqing, 400042 China

**Keywords:** Intra-abdominal volume increment, Intra-abdominal hypertension, Trauma, Shock, Resuscitation

## Abstract

**Background:**

To investigate the effect of intra-abdominal volume increment (IAVI) on intra-abdominal hypertension (IAH) in the kidneys.

**Methods:**

Eight minipigs were successfully established as IAH models and were randomly divided into two groups: the IAVI group and the sham-operated group. The intravesicular pressure, inferior vena cava pressure and urine volume were measured before shock, 2 h after IAH, and 22 h after surgery, respectively. The following indices were measured: serum creatinine, urea nitrogen, renal cortical thickness, ratio of abdominal anteroposterior diameter/transverse diameter, renal thickness, diameter of the renal sinus and the wet/dry ratio of renal tissues.

**Results:**

The intravesicular pressure (IVP) of the 8 minipig IAH models was calculated to be 21.16 ± 4.63 mmHg. There was a significant increase in the abdominal anteroposterior diameter/transverse diameter ratio. The minipigs in the IAVI group survived during the observational period, whereas 2 minipigs died at 18 h and 20 h in the sham-operated group. Twenty-two hours after surgery, the animals in the IAVI group displayed increased urinary volume (UV) and decreased Cr and Ur and remarkable decreases of VP and IVCP. After IAH, the renal cortical thickness and the renal thickness increased significantly. The renal wet/dry ratio in the sham-operated group was higher than that in the IAVI group.

**Conclusion:**

IAVI helps to control renal dysfunction after IAH, which may be related to lowering the intra-abdominal pressure, thus alleviating renal edema and blood stasis.

## Background

An intra-abdominal pressure (IAP) exceeding 12 mmHg is considered to be pathologically elevated and is referred to as intra-abdominal hypertension (IAH). This condition, which results from pancreatitis, trauma, capillary leakage, temporary abdominal packing, and visceral edema, may lead to the abdominal compartment syndrome (ACS) [1].

Early studies indicated that IAH might elevate renal venous pressure, increase renal parenchymal pressure, and decrease renal blood flow and glomerular filtration rate, resulting in oliguria or anuria [2]. Renal hypoperfusion and renal parenchyma compression can activate the renin-angiotensin-ketosteroid system and subsequently induce water and sodium retention [3]. This action further increases vascular resistance and may lead to a vicious cycle. Previous studies have demonstrated that increased intra-abdominal pressure resulted in both obstructive and non-obstructive impairment of renal pelvic excretion [4].

Treatment for IAH includes both surgical and non-surgical modalities; however, the optimal surgical program has not been established. Closure assisted by vacuum sealing drainage (VSD) is currently the most commonly used strategy for temporary abdominal closure. Intra-abdominal volume increment (IAVI) is a technique that we have modified for treating IAH; additional studies concerning the effect of the abdominal increment surgeries on renal function are required, as are imaging studies comparing the tissue before and after surgery. The present study established an IAH model in large animals by incompletely blocking the portal vein to induce IAH and investigated the effect of IAH on kidneys after hemorrhagic shock and resuscitation and the therapeutic effect of IAVI in improving the success rate of clinical treatment.

## Methods

### Animals

This study was approved by the Third Military Medical University Council on Animal Care in accordance with the guidelines of the Ministry of Science and Technology of People’s Republic of China (The Guidance of Experimental Animal Welfare, 2006).

Twelve healthy minipigs (Bama, Guangxi) (provided by the Experimental Animal Center in Daping Hospital of the Third Military Medical University) with an average weight of 22.9 ± 1.7 kg were used.

### Reagents and instrument

Laboratory reagents included normal saline (0.9% NaCl, 500 mL; Sichuan Kelun Pharmaceutical Co., Ltd., Chengdu, China), Ringer’s solution (NaCl 0.85%, KCl 0.03%, CaCl_2_ 0.033%, 500 mL; Sichuan Kelun Pharmaceutical Co., Ltd., Chengdu, China), KCl (10 mL:1 g; Wubei Tiansheng Kangdi Pharmaceutical Co., Ltd., Wuhan, China), ketamine (2 mL:0.1 g; Jiangsu Hengrui Medicine Co., Ltd., Lianyungang, China), and pentobarbital sodium (25 g, P3761; Sigma-Aldrich, San Francisco, CA, USA). The equipment used included a blow-extruded double blood bag (Jiaxing Tianhe Pharmaceutical Co., Ltd., Zhejiang, China), single-lumen catheters (SWL-1996-01C-03-20; Sungwon, Korea), and polyvinyl alcohol gelfoam foam material (Weisidi Medical Technology Co., Ltd, Wuhan, China). Instruments that were used included an anesthesia machine (SOUSAR; Beijing Yian Medical Inc., Beijing, China), a multifunction monitor (M3046A; Hewlett-Packard, Palo Alto, CA, USA), a Vigileo monitor (American Edwards Lifesciences Corporation, Irvine, CA, USA), a blood gas analyzer (GEM Premier 3000; American Society for Testing Instruments, Irvine, CA, USA), and a CT scanner (LightSpeed VCT, General Electric Company, NY, USA).

### Establishment of IAH model after hemorrhagic shock and resuscitation

We used the model modified by Shah et al. [5, 6]. The animals were fasted for 12 h before surgery with free access to water and were anesthetized by posterior auricular vein injection of ketamine (7.2 mg/h/kg) and 3% sodium pentobarbital (6 mg/h/kg). The animals were placed on the experimental table, followed by skin disinfection.

After tracheotomy, assisted respiration was performed using an anesthesia machine with pure oxygen inhalation. The right femoral artery and vein were isolated, and single lumen catheters were inserted. The catheter was connected, via a triplet, to the transducer and multifunctional monitor. The catheterized artery was used for bleeding, whereas the catheterized vein was used for fluid transfusion and pressure detection in the inferior vena cava. After midline abdominal incision, a cystostomy was performed. The portal vein was isolated and ligated using a hard plastic tube (with an external diameter of 5 mm) and a silk line. The hard plastic tube was removed, creating a model of incomplete blockage of the portal vein to simulate the process of perihepatic packing for the treatment of serious hepatic injury.

Femoral artery exsanguination was performed until the mean arterial pressure (MAP) reached 50 mmHg, and the blood was stored in the blood bags. The abdominal wall incision was closed by layered sutures. One hour after shock, all of the lost blood, along with Ringer’s solution (twice the amount of the blood), was transfused. Ringer’s solution was continuously administered to achieve over-resuscitation. The IAH model was considered to be successfully established when an IAP of ≥12 mmHg was maintained for 1 h [7]. Before the model was successfully established, 4 animals died of hypovolemic shock.

### Animal groups and phase-point observations

Four animals that died before the successful establishment of the model were excluded from the study. Using a coin-toss method, the remaining 8 animals were divided into the IAVI group (*n* = 4) and the sham-operated group (*n* = 4). The IAVI treatment procedure was performed as follows [8]: removing the abdominal incision sutures, extending the incision from the xiphoid to the pubic symphysis, loosening and removing the ligatures of the portal vein, and flattening the omentum above the bowels. A polyvinyl alcohol gelatin foam sponge of approximately 30 × 20 cm was placed over the omentum and sew the full layer of the abdominal wall. The drainage tube embedded in the sponge was extracted from one side of the incision. A biological permeable membrane with good oxygen and moisture permeability was applied to cover the incision and the sponge until the incision is sealed. Drainage tubes were used to maintain a negative pressure of 60 mmHg to 80 mmHg with continuous suction until the animals were killed. In the sham-operated group, it was to remove the abdominal incision sutures, extend the incision from the xiphoid to the pubic symphysis, loosen and remove the ligature of the portal vein and directly close the incision using layered sutures.

Measurements were taken before shock, 2 h after the initiation of IAH and 22 h after surgery. The animals were sacrificed using intravenous injection of 0.2 g ketamine and 10 ml of 10% potassium chloride.

#### Measurement indexes of renal function

Urinary volume (UV) was recorded per hour, and blood samples were drawn using the inferior vena cava catheter to measure the creatinine and urea nitrogen levels (after the blood sample had clotted, a serum sample was collected for analysis using the DXC800 automatic biochemical analyzer) before shock, 2 h after the initiation of IAH, and 22 h after surgery.

### Measurements of indexes affecting renal function

The following measurements were taken before shock, 2 h after the initiation of IAH, 22 h after surgery, and 26 h after surgery. (1) Intravesicular pressure (IVP): After the bladder was evacuated, 50 ml of normal saline was injected into the bladder, and the silicone tube was erected; the vertical height of the water column above the pubic symphysis was taken as the intravesicular pressure when the column declined to a steady level. (2) Inferior vena cava pressure (IVCP): IVCP was measured using a multi-function monitor via the inferior vena cava catheter. (3) Color Doppler: An ultrasound examination was performed by measuring the thickness of the renal cortex, observing whether there was separation of the renal pelvis and the renal calyx, based on the data obtained from the right kidney. (4) Enhanced CT scan: The ratio of the anteroposterior diameter of the abdomen to the transverse diameter was measured (at the level of the left renal vein, the subcutaneous fat was removed) [9]; renal thickness and renal pelvis length at the widest cross-section of the kidney were measured, based on the data obtained from the right kidney. (5) Wet/dry ratio of renal tissues: The upper 1/8 of the left kidney tissue was extracted, the kidney specimen were weighed and placed in a 60°C drying oven for 3 days. A wet/dry ratio of 4-4.5 or higher indicated severe organ edema. (6) Kidney histopathology: The upper 1/8 of the right kidney tissue was extracted and fixed in 10% formalin, embedded in paraffin, sliced, performed hematoxylin-eosin (HE) staining, and observed under light microscopy.

### Statistical analysis

All data, expressed as the mean ± SD values, were analyzed using SPSS 17.0 software and Student’s *t* test for comparing the mean values among the groups. *P* < 0.05 was considered to be statistically significant.

## Results

### IAH model

**Figure 1:**
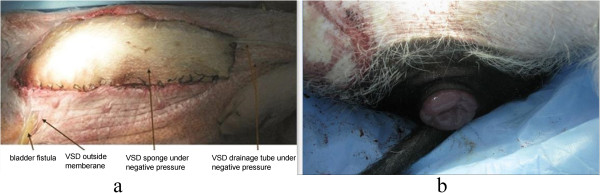
**Photos of IAH model. a**. Abdominal incision after IAVI therapy; **b**. rectal prolapse after IAH.

The average body weight was 22.90 ± 1.70 kg and baseline MAP was 118.00 ± 14.30 mmHg. Exsanguination was performed until an MAP of 50 mmHg was achieved; exsanguination volume was 648.00 ± 98.00 ml. The baseline IVP was 6.65 ± 0.50 mmHg. When IVP reached 21.16 ± 4.63 mmHg for 3.20 ± 0.60 h, the required amount of Ringer’s solution was 76.00 ± 3.00 mL/kg/h. Two (25%, maximum IVP of 17.6 0 mmHg and 19.20 mmHg, respectively) minipigs exhibited rectal prolapse and stress urinary incontinence (Figure 1).

The IAH model was successfully established in 8 (66%) of 12 minipigs. In the IAVI group, all four minipigs survived during the observation period. In the sham-operated group, 2 of 4 pigs died because of respiratory failure at 18 h and 20 h, respectively, after the initiation of IAH.

UV at 2 h after the initiation of IAH was significantly less than that before shock (55 ± 11 ml/h *vs.* 156 ± 35 ml/h, *P* < 0.01). In the IAVI group, urinary volume recovered (120 ± 23 ml/h *vs.* 55 ± 11 ml/h, *P* < 0.01) but did not reach normal level (120 ± 23 ml/h *vs.* 156 ± 35 ml/h, *P* < 0.05). Urinary volume was significantly lower in the sham-operated group compared to the IAVI group (10 ± 135 ml/h, *P* < 0.01).

Blood Cr and Ur values increased at 2 h after the initiation of IAH, without significant difference (6.5 ± 8.5 μmol/L *vs.* 72.3 ± 6.1 μmol/L; 3.87 ± 1.05 mmol/L *vs.* 4.12 ± 0.85 mmol/L, *P* > 0.1). After the IAVI treatment, Cr and Ur continued to increase slowly, without significant change in Ur (4.73 ± 0.71 mmol/L *vs.* 4.12 ± 0.85 mmol/L, *P* > 0.1; 89.5 ± 9 μmol/L *vs.* 72.3 ± 6.1 μmol/L, *P* < 0.05). In the sham-operated group, both Cr and Ur were significantly higher than the values in the other groups (200 ± 1.4 μmol/L, 9.6 ± 0.14 mmol/L, *P* < 0.01) (Table 1).

**Table 1:** **Effect of IAH and post-surgery protocol on renal function**

Index	Group	Before shock (***n*** = 8)	2 h after IAH (***n*** = 8)	22 h after surgery (***n*** = 4)
Urinary volume (ml/h)	IAVI group	156.00 ± 35.00	55.00 ± 11.00^§^	120.00 ± 23.0^★△^
	Sham-operated group			10.00 ± 1.00
Cr (μmol/L)	IAVI group	66.50 ± 8.50	72.30 ± 6.10^§^	89.50 ± 9.00^△^
	Sham-operated group			200.00 ± 1.400
Ur (mmol/L)	IAVI group	3.87 ± 1.05	4.12 ± 0.85^§^	4.73 ± 0.72^△^
	Sham-operated group			9.60 ± 0.140
IVP (mmHg)	IAVI group	6.65 ± 0.5	21.16 ± 4.63	10.38 ± 0.99
	Sham-operated group			31.65 ± 3.04
IVCP (mmHg)	IAVI group	6.59 ± 0.52	21.15 ± 4.59	10.4 ± 1.14
	Sham-operated group			32 ± 2.82

Compared with before shock, ^§^P < 0.05. Compared with 2 h after IAH, ^★^P < 0.05. IAVI group *vs.* sham-operated group, ^△^P < 0.05.

The wet/dry ratio of renal tissues in the sham-operated group was remarkably higher than that in the IAVI group (5.8 ± 0.7 *vs.* 3.7 ± 0.6, *P* < 0.01).

### Relevant factors affecting renal function

Two hours after IAH, IVP and IVCP were obviously higher than those in pre-shock (21.16 ± 4.63 mmHg *vs.* 6.65 ± 0.5 mmHg, 21.15 ± 4.59 mmHg *vs.* 6.59 ± 0.52 mmHg, *P* < 0.01). Twenty-two hours after IAVI treatment, IVP and IVCP showed a significant decrease after IAH (10.38 ± 0.99 mmHg *vs.* 21.16 ± 4.63 mmHg, 10.4 ± 1.14 mmHg *vs.* 21.15 ± 4.59 mmHg, *P* < 0.01), which were slightly higher than those in pre-shock, but did not reach significant difference (10.38 ± 0.99 mmHg *vs.* 6.65 ± 0.55 mmHg, 10.4 ± 1.14 mmHg *vs.* 6.59 ± 0.52 mmHg, *P* > 0.1). IVCP and IVP were significantly higher in the sham-operated group compared to the IAVI group (32 ± 2.82 mmHg *vs.* 31.65 ± 3.04 mmHg, *P* < 0.01). The correlation coefficient between IVP and IVCP was 0.99 (*P* < 0.01) (Table 1).

### Imaging changes

**Figure 2:**
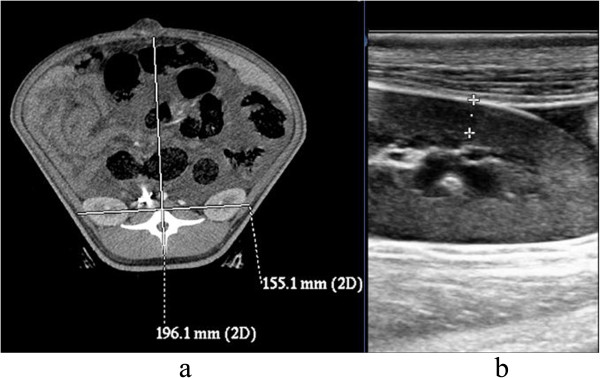
**CT and ultrasonic images of kidney. a**. Round belly after ACS (the anteroposterior diameter/transverse diameter ratio was 1.26); **b**. ultrasonic imaging of the renal cortex.

The ratio of the anteroposterior diameter to the transverse diameter was calculated (including the data for animals that died within 2 h after the initiation of IAH (n = 2)). The ratio at 2 h after the initiation of IAH was significantly higher than the baseline ratio (1.22 ± 0.14 *vs.* 0.96 ± 0.01, *P* < 0.01) (Figure 2).

The renal height of the right kidney was measured using CT, which indicated no significant difference between the IAVI group and the sham-operated group. The height in the sham-operated group was higher than that in the other groups (19 ± 1.4 mm *vs.* 12.5 ± 4.2 mm, 12.5 ± 4.2 mm*,* 15.7 ± 1.8 mm, *P* < 0.05). The renal cortical thickness in the right kidney was measured using B ultrasound, which demonstrated no significant difference between the IAVI treatment group and the sham-operated group. The thickness in the sham-operated group was higher compared to the other groups (9.3 ± 0.4 mm *vs.* 5.5 ± 0.8 mm, 6.8 ± 1.0 mm, 5.9 ± 0.7 mm, *P* < 0.05) (Table 2).

**Table 2:** **Thickness measured by CT and B ultrasound**

Index (mm)	Group	Before shock (***n*** = 8)	2 h after IAH (***n*** = 8)	22 h after surgery (***n*** = 4)
Right kidney thickness as determined by CT	IAVI group	12.50 ± 4.20	13.50 ± 3.20^§^	15.70 ± 1.80^★△^
	Sham-operated group			19.00 ± 1.40
Right renal cortical thickness as determined by B ultrasound	IAVI group	5.50 ± 0.80	6.80 ± 1.00	5.90 ± 0.70^△^
	Sham-operated group			9.30 ± 0.40

Compared with before shock, ^§^P < 0.05. Compared with 2 h after IAH, ^★^P < 0.05. IAVI group *vs.* sham-operated group, △P < 0.05.

The renal sinus diameter of the right kidney was measured using CT. There was no obvious change in IAH (41.4 ± 5.7 mm *vs.* 41.3 ± 5.5 mm, *P* > 0.1). There was no hydronephrosis or separation of the renal pelvis and the renal calyx, as determined by B ultrasound and CT, at any phase point in all of the animals.

### Pathological results

**Figure 3:**
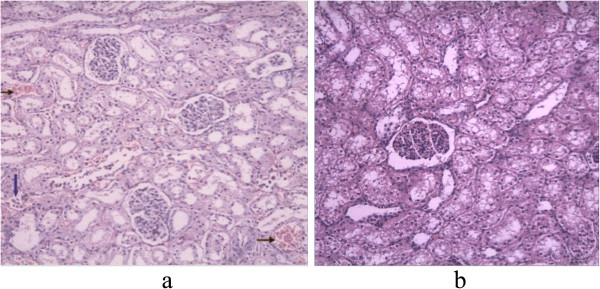
**Histomorphological images of kidney. a**. Sham-operated group revealed moderate blood stasis in the kidney accompanied by the infiltration of inflammatory cells. The horizontal arrows indicated the red blood cells, and the vertical arrow indicated the inflammatory cells (HE staining, 200×); **b**. there was no abnormality (HE staining, 200×).

In the sham-operated group, obvious blood stasis was observed in the kidney accompanied, by the infiltration of inflammatory cells. In the IAVI group, only mild blood stasis or no blood stasis and slight infiltration of inflammatory cells were observed (Figure 3).

## Discussion

In the present study, we observed a decrease of UV and slight increases in the Cr and Ur levels at 2 h after the initiation of IAH. At 22 h, notable oliguria and pathological damage had occurred. These results were consistent with the outcomes of previous studies [2, 9], which reported that oliguria was observed when IAP reached 15-20 mmHg, anuria was observed when IAP was higher than 30 mmHg, and histological changes, such as renal tubular necrosis, were observed when IAP reached 15 mmHg.

Bingol-Kologlu et al. [4] reported that the increases in IAP may impair the passage of urine from the renal pelvis through the compressive effect upon ureters together with a concomitant increase in intravesical pressure. In the present study, hydronephrosis and separation of the renal pelvis and the renal calyx were not observed using CT or color Doppler ultrasound. Whether the decline in UV was largely determined by this factor requires further investigation. It is not easy to distinguish between IAH/ACS-induced oliguria and insufficient fluid resuscitation [10]. Once IAH-induced oliguria is not recognized, a persistent increase in the amount of fluid resuscitation will lead to serious consequences [11].

Because IAH is harmful, it is important to explore suitable therapies. There are non-surgical and surgical treatment options. Opening the abdomen, which is the most effective method for reducing IAP, is the treatment of choice for ACS when IAP is constantly higher than 30 mmHg with ongoing organ failure refractory to medical therapy [12]. Recent research has indicated that prophylactic laparotomy could increase the survival rate for patients with IAH/ACS. The open abdominal approach is now commonly used after ACS instead of the abdomen management approach [13–15]. Decompression seems to have a beneficial effect on renal dysfunction, although there are conflicting data [16]. There are many approaches for ACS surgical treatment [17–19], such as the pure skin suture technique and vacuum-assisted closure (VAC). Presently, VAC is widely applied. The negative pressure device, which is the most effective strategy for temporary abdominal closure, has a higher rate of primary fascial closure and a lower risk of fistulas [12, 20, 21]. The expansion technique that we applied is the modified VAC technique. However, several studies did not recommend the VAC technique immediately after decompressive laparotomy because it did not provide sufficient capacity to further increase abdominal volume and, therefore, might pose an increased risk for the recurrence of IAH [22]. However, the aim of our study is not to study the model with serious ACS but to investigate the therapeutic effect of a surgery similar to prophylactic surgery, which uses effective expansion to relieve the pressure on the internal organs and to achieve peritoneal exudate drainage [23, 24]. Finally, we observed a satisfying therapeutic effect during the observation period. The data on renal thickness and cortex thickness, pathological findings, UV, Ur and Cr and renal water content in the IAVI group demonstrated improvement compared with those in the sham-operated group, indicating the effectiveness of IAVI treatment.

IAP monitoring is crucial for patients with IAH and ACS [24]. The gold standard for IAP monitoring is the measurement of IVP. However, in cases of associated pelvic fracture, bladder perforation, tumor, or neurogenic bladder, alternative methods must be considered for monitoring abdominal pressure [9, 25–29]. Our studies observed that 50 ml of normal saline injected into the bladder is excreted through the urethra, similar to the condition of stress incontinence. This action leads to a lower measured level compared to the actual level, which requires physicians to pay more attention to avoid missing the diagnoses of IAH and ACS. Meanwhile, our experiment demonstrated a good correlation coefficient of 0.99 between the inferior vena cava pressure and the bladder pressure. Hence, the inferior vena cava pressure can be used as a supplementary means of measurement, if necessary.

Al-Bahrani et al. [9] proposed seven indexes to predict IAH and ACS. Two indices had practical significance: one index was the round belly sign (defined as transverse diameter > 0.8, measured at the level where the left renal vein crosses the aorta and excluding the subcutaneous fat); the other index was gastrointestinal wall thickening. Renal compression, displacement or intra-abdominal parenchyma pressure or organ displacement (contour deformity) and bilateral inguinal hernia are not meaningful in the patient’s clinical evaluation. In the minipigs we studied, the normal anteroposterior diameter/transverse diameter ratio was 0.95, which increased to 1.16 ± 1.47 after IAH, indicating a significant difference. In the present study, the position of the kidney did not shift or contract due to pressure. However, renal thickness (confirmed by both B ultrasound and CT) increased significantly due to renal blood stasis and edema. Moreover, 2 minipigs had rectal prolapse, representing internal organ swelling due to increased IAP. The significance may be similar to that of abdominal external hernia. Malbrain et al. [30] indicated a poor correlation between abdominal circumference and intra-abdominal pressure. The abdominal circumference could not be used to quantify the intra-abdominal pressure. Thus, a positive sign on CT is only a supplement to IAH/ACS and can never replace IAP monitoring. The relationship between abdominal circumference and CT anteroposterior diameter requires further investigation.

Renal dysfunction in IAH is a multifactorial process, and the involved mechanisms have not been established. Elevations of the renal parenchymal and renal vein pressure had been suspected as the possible mechanisms for renal impairment in IAH/ACS [31, 32]. Although an isolated increase in parenchymal pressure may not be sufficient to cause renal dysfunction, it contributes to the acute kidney injury caused by the ischemic insult. The isolated elevation of renal vein pressure will result in a significant decrease in glomerular filtration rate, with a significant increase in serum aldosterone levels and plasma renin activity [32, 33]. Our study confirms that blood stasis and infiltration of inflammatory cells lead to elevated renal thickness with the increase in IAP. IAVI can rapidly decrease IAP and maintain this parameter at a low level, which could alleviate the compression of the kidney and relieve the renal congestion and edema, thereby improving renal function.

Although the improvement provided by expansion therapy is not obvious over the short term (22 h), the improvement in renal function is obvious. Thus, preventative expansion surgery is a good approach for treating IAH. Regarding the study of ACS, despite some progress, there are still many unknown aspects to explore. There are several limitations in our study: (1) small sample size and many confounding factors; (2) high mortality in the animal models and inaccurate control of the value of IAP; and (3) short observation time. All of these disadvantages warrant additional investigation.

## Conclusions

IAH/ACS frequently occur in critical patients and may affect various organ systems, and kidney is one of the most often affected organs. A correlation between reduction in IAP induced by IAVI and improvement in renal function was observed. IAVI helps to control renal dysfunction after IAH, which may be related to lowering the intra-abdominal pressure, thus alleviating renal edema and blood stasis.
